# Visceral leishmaniasis (kala-azar) outbreak in Somali refugees and Kenyan shepherds, Kenya.

**DOI:** 10.3201/eid0707.010746

**Published:** 2001

**Authors:** G Boussery, M Boelaert, J van Peteghem, P Ejikon, K Henckaerts


### Visceral Leishmaniasis (Kala-Azar) Outbreak in Somali Refugees and Kenyan Shepherds, Kenya

**To the Editor:** A sharp increase in suspected visceral leishmaniasis (VL or kala-azar) cases was reported in April through May 2000 in three Kenyan refugee camps (Ifo, Dagahaley, and Hagadera). Located around Dadaab town in Northeastern Province, the three camps house an estimated 125,000 Somali refugees. VL outbreaks have been well documented in five distinct foci in Kenya (1,2), but until this outbreak, VL was only sporadically seen in the refugee camps or the province.

We investigated a possible outbreak in the refugee sites. Before April 2000, doctors would request a formol-gel test (FGT) in case of suspected VL and treat an FGT-positive case with antimonials. Although the FGT is of uncertain validity, it is still used in district hospitals in Kenya for lack of alternative diagnostic tests. We considered a clinician's request of an FGT as a proxy for "clinical VL suspicion" and assessed the number of FGTs done from January 1999 to March 31, 2000. The first suspected VL patient was traced back to August 1999; this 40-year-old male Somali refugee had been ill for 8 months and sought treatment at Dagahaley camp. He responded well to antimonial treatment. From that date to April 1, 2000, an FGT was requested for five more patients; results were positive for two.

Specific surveillance for VL was set up by the refugee health services in April 2000. Suspected patients or their caretakers were interviewed. Finger-prick blood was collected on filter paper and analyzed by direct agglutination test (DAT) (3). In August 2000, splenic aspirates were performed on eight patients for direct microscopic examination, and parasite culture was attempted for three specimens. In vitro isolation and gp63 polymerase chain reaction-restriction fragment length polymorphism (PCR-RFLP) molecular typing was done at the Protozoology Unit of the Prince Leopold Institute of Tropical Medicine in Antwerp, Belgium. Serologically or parasitologically confirmed cases were given stibogluconate (Pentostam), 20 mg/kg/day, for 28 days.

We reviewed surveillance data for the period April 1–August 31, 2000, and interviewed the health staff. For case classification, a probable case of VL was illness in a patient with 1) a fever of >2 weeks' duration, 2) splenomegaly or wasting, and 3) positive DAT serology. A confirmed case had these clinical signs, as well as a positive parasitology smear or culture. From April 2000 to August 31, 2000, 26 probable (DAT-positive) VL cases were observed and 8 others were confirmed parasitologically. Gp63 PCR-RFLP molecular typing showed *Leishmania donovani* in one specimen. The case-fatality rate was 10 (29.4%) of 34 patients in the group of probable and confirmed VL cases. Six deaths occurred before treatment could be started, and one was a complication of the diagnostic procedure (spleen aspirate).

Thirty-two interviews were completed in the group of 34 probable or confirmed VL patients. Median age was 15 years, and 8 (25) of the 32 were female. Median delay between onset of symptoms and date of diagnosis was 8 months. Six were Kenyan citizens, five of them shepherds who were grazing their cattle in the area around Dadaab. Of the Somali refugees, seven had been living for >2 years in the refugee camp when their symptoms began. (Five had been born in the camps.) However, 16 (61.5%) of 26 patients arrived in the camps after the onset of their symptoms. Most of them were Ogadeni shepherds, who reportedly grazed their cattle in the Lower Juba region.

Other evidence points to a serious problem inside Somalia as well. Médecins sans Frontières reported 48 VL cases from July 28 to September 21, 2000, in Hudur, Bakol region. Other nongovernmental organizations reported cases from several towns in Gedo region. The distribution of VL in Somalia before the war is poorly documented, but the disease was known to be endemic in Giohar district, north of Mogadishu (4). In 1994, Woolhead reported VL in a woman from Baidoa and warned of potential outbreaks because of the war (5). Although several of the 34 patients reported here may have been infected in Somalia, local transmission in Kenya cannot be excluded, since some of the refugees denied having left camp and six were Kenyan citizens.

This outbreak is reason for concern in the context of the deteriorating nutritional situation in drought-affected northeastern Kenya. Malnutrition is a known risk factor for the development of clinical VL in infected persons (6). In southern Sudan, deaths caused by VL were attributed to malnutrition in a famine- and war-stricken population (7). The current nutritional status of the Somali refugees in the Dadaab camps is precarious. After the 1996 food scarcity problem (8), food rations for refugees were maintained at the recommended 2,100 kcal/person/day. In February 2000, however, the ration was again reduced below the vital minimum. A cross-sectional random cluster survey on August 29-31, 2000, showed rising malnutrition levels in <5-year-old refugee children (Médecins sans Frontières, unpub. data).

Immediate outbreak control measures have been taken by refugee camp health authorities, the surveillance system was strengthened (including initiation of active case-finding measures), and diagnostic and therapeutic facilities were upgraded. Six-month peridomestic spraying of the refugee shelters with lambda-cyhalothrin (ICON) is a routine vector control measure in the camps. Special attention needs to be paid, however, to the food security for the refugees in the light of the current outbreak. Our observations on 16 imported cases also raise concerns about VL transmission inside Somalia, where access to health care is virtually nonexistent in many areas and a VL outbreak might go undetected.

### Acknowledgment

We thank the doctors and nurses in the Dadaab refugee health services for data collection and J.C. Dujardin for species identification.

**Günter Boussery,* Marleen Boelaert,† Joke Van Peteghem,* Philip Ejikon,‡ and Koen Henckaerts***

*Médecins sans Frontières, Brussels, Belgium; †Institute of Tropical Medicine, Antwerp, Belgium; and ‡Médecins sans Frontières, Nairobi, Kenya

### References

1. Schaefer KU, Kurtzhals JA, Sherwood JA, Githure JI, Kager PA, Muller AS. Epidemiology and clinical manifestations of visceral and cutaneous leishmaniasis in Baringo District, Rift Valley, Kenya. A literature review. Trop Geogr Med 1994;46:129-33.

2. World Health Organization. Control of the leishmaniases. Report of a WHO Expert Committee. World Health Organ Tech Rep Ser 1990;793:1-158.
3. El Harith A, Kolk AH, Kager PA, Leeuwenburg J, Muigai R, Kiugu S, et al. A simple and economical direct agglutination test for serodiagnosis and sero-epidemiological studies of visceral leishmaniasis. Trans R Soc Trop Med Hyg 1986;80:583-7.
4. Shiddo SA, Aden Mohamed A, Akuffo HO, Mohamud KA, Herzi AA, Herzi Mohamed H, et al. Visceral leishmaniasis in Somalia: prevalence of markers of infection and disease manifestations in a village in an endemic area. Trans R Soc Trop Med Hyg 1995;89:361-5.
5. Woolhead A. A recent case of visceral leishmaniasis in Somalia. Ann Trop Med Parasitol 1995;89:687-8.
6. Cerf BJ, Jones TC, Badaro R, Sampaio D, Teixeira R, Johnson WD Jr. Malnutrition as a risk factor for severe visceral leishmaniasis. J Infect Dis 1987;156:1030-3.
7. Seaman J, Mercer AJ, Sondorp E. The epidemic of visceral leishmaniasis in western Upper Nile, southern Sudan: course and impact from 1984 to 1994. Int J Epidemiol 1996;25:862-871.
8. Boelaert M, Englebert M, Hanquet G, Van Damme W, Van der Stuyft P. Refugee relief rations. Lancet 1997;349:1775.

### Doxycycline and Eradication of Microfilaremia in Patients with Loiasis

**To the Editor:** *Wolbachia* are intracellular symbionts found in 20% of insects and in several nematodes, including filarial worms. Because tetracycline eradicates *Wolbachia* in nematodes, this drug has been proposed for chemotherapy in filariasis (1). We report two patients with loiasis in whom no Wolbachial DNA was detected in microfilariae by polymerase chain reaction (PCR), and for whom 6 weeks of doxycycline failed to eradicate the microfilaremia. We conclude that doxycycline may not be an efficient therapy for loiasis.

Filariae are responsible for 150 million infections worldwide, some of them devastating diseases such as elephantiasis (caused by Bancroftian and Brugian filariasis) and blindness (caused by onchocerciasis). There is no satisfactory treatment for filariasis: although diethycarbamazine citrate has been used for 50 years to treat the disease, this drug is not efficient in most adults (2). Ivermectin has been reported to be efficient in treating microfilaremia and possibly for viability and fertility of adult *Oncocerca volvulus* worms if therapy is prolonged for 2 to 3 years (2). However, the microfilaricidal effect of ivermectin on *Wuchereria*, *Brugia*, and *Loa loa* is similar to diethycarbamazine: ultrasonography shows that it has no effect on adult Bancroftian worms, and microfilariae often reappear after a few months (2).

Intracellular bacteria have been observed in the lateral cords of adult female worms as well as in microfilariae of *W. bancrofti,* and these bacteria have recently been identified as belonging to the *Wolbachia* genogroup (3). *Wolbachia* have been detected by PCR in Brugian and Bancroftian filariae, dirofilariae, and most species of *Oncocerca* including *O. volvulus*, but have never been detected in *L. loa* worms (4). *Wolbachia* are causative agents of a variety of modifications in host development and reproduction, including cytoplasmic incompatibility and parthenogenesis. Consequently, it has been proposed that antibiotic eradication of *Wolbachia* from infected filarial worms would reduce microfilaremia. This has been demonstrated in an animal model with *Litomosoides sigmodontis* and recently confirmed in patients infected with *O. volvulus* (1-5). In experimental *L. sigmodontis* infection, after 41 days of therapy, microfilaremia in tetracycline-treated animals was one-tenth that in normally infected animals (5).

We report the failure of tetracycline to reduce microfilaremia in two patients with *L. loa* filariasis. Patient no. 1 was a 58-year-old man who worked in Gabon for many years and had cutaneous larva migrans. *L. loa* microfilaremia was detected ($4x10^3$/mL). Patient no. 2, a 15-year-old boy living in Cameroon, was diagnosed with Calabar swelling with *L. loa* microfilaremia ($1x10^3$/mL). After giving informed consent, both patients were treated with doxycycline 200 mg daily for 6 weeks as previously described in *O. volvulus*-infected patients (1). We observed patients for microfilaremia every week for 6 weeks and then every 2 weeks for 2 months. The presence of adult worms was detected by physical examinations.

Microfilaremia was detected in both patients at the completion of treatment and at day 120 of follow-up. In patient no. 1, the frequency of migrating adult worms seemed to diminish during therapy, but they never disappeared. For *Wolbachia* detection in worms, blood samples were collected both in Dupont-Isolator and EDTA-containing tubes. After centrifugation at 5000 x *g* for 30 min, the worm-enriched pellet was resuspended in 1 mL of sterile deionized water for erythrocyte lysis. DNA was extracted from the suspension by using the QIAmp-blood kit (Qiagen, Hilden, Germany) following manufacturer's recommendations. *W. pipientis* DNA was used as a positive control. Control of DNA extraction was performed by amplifying microfilarial DNA using the nematode-specific 18S r DNA-derived primers 18SF (5'-GAT-ACC-GCC-CTA-GTT-CTG-ACC-3') and 18SR (5'-ACC-AAC-TAA-GAA-CGG-CCA-TG-3'). *Wolbachia* detection was attempted with the FD1 (5'-AGA-GTT-TGA-TCC-TGG-CTC-AG-3') and Rp2 (5'-ACG-GCT-ACC-TTG-TTA-CGA-CTT-3') eubacterial primers, with the *Ehrlichia* genus-specific 16S rDNA primers EHR16SD (5'-GGT-ACC-YAC-AGA-AGA-AGT-CC-3') and EHR16SR (5'- TAG-CAC-TCA-TCG-TTT-ACA-GC-3'), and with primers specific for the 16S r DNA of *B. malayi* endosymbiont, Bsymbf (5'-ACG-AGT-TAT-AGT-ATA-ACT-3'), and Bsymbr (5'-CCT-TCG-AAT-AGG-AAT-AAT-3') (3-6). PCR reactions were performed on PTC-200 thermocycler (MJ-Research, USA) by using 45 cycles of denaturation at 94°C for 30 sec, hybridization for 45 sec, and elongation at 72°C for 1 min. Hybridization temperatures were 55°C for FD1/Rp2, 53°C for EHR16SD/ EHR16SR, 42°C for Bsymbf/Bsymbr, and 57°C for 18SF/18SR. Experiments were repeated three times.

We detected *Wolbachia* in the positive control and 18s rRNA of the nematode in the sample, but no signal compatible with Wolbachial DNA was obtained with the sets of primers used. In fact, four species of filariae *(Dipetalonema setariosum*, *Acanthocheilonema vitae*, *O. flexuosa*, and *L. loa*) tested for intracellular bacteria by electron microscopy, immunohistochemistry, or PCR had no bacteria (4). The absence of *Wolbachia* in *L. loa* microfilariae may explain the failure of tetracycline therapy in our patients.

More work is needed to determine the prevalence of *Wolbachia* in filariae, their impact on fertility in each species, and the use of antibacterial agents for eradicating these pathogens.

**Acknowledgment**

We thank MJ Taylor for providing *W. pipientis* DNA for controls.

